# The effect of a music-based care intervention on neuropsychiatric symptoms: secondary analysis of a cluster-randomised controlled study in nursing home residents with dementia and pain

**DOI:** 10.1186/s12877-026-07301-4

**Published:** 2026-03-19

**Authors:** Martin Elstad Myrenget, Reidun Sandvik, Petter Borchgrevink, Geir Selbæk, Milada Småstuen, Vegar Rangul, Odd Håpnes, Audun Myskja, Bettina Husebø, Tone Rustøen

**Affiliations:** 1 Department of Circulation and Medical Imaging, Faculty of Medicine, Norwegian University of Science and Technology, Trondheim, Norway; 2 Laugsand Helse- Og Velferdssenter, Trondheim Municipality, Trondheim, Norway; 3 Centre for Care Research, West, Western Norway University of Applied Sciences, Bergen, Norway; 4 Department of Health and Caring Sciences, Faculty of Health and Social Sciences, Western Norway University of Applied Sciences, Bergen, Norway; 5 Department of Pain and Complex Disorders, Clinic of Anesthesia and Intensive Care, St. Olav’s Hospital, Trondheim, Norway; 6 Norwegian National Centre for Ageing and Health, Vestfold Hospital Trust, Tønsberg, Norway; 7 Department of Geriatric Medicine, Oslo University Hospital, Oslo, Norway; 8 Faculty of Medicine, University of Oslo, Oslo, Norway; 9 Department of Research and Development, Division of Emergencies and Critical Care, Oslo University Hospital, Oslo, Norway; 10 Institute of Health and Society, Faculty of Medicine, University of Oslo, Oslo, Norway; 11 Norwegian Resource Centre for Arts and Health, Nord University, Faculty of Nursing and Health Sciences, Levanger, Norway; 12 Department of Public Health and Nursing, HUNT Research Centre, Faculty of Medicine and Health Sciences, Norwegian University of Science and Technology, Levanger, Norway; 13 Senter for Livshjelp, Ski, Norway; 14 Department of Global Public Health and Primary Care, Centre for Elderly and Nursing Home Medicine, Faculty of Medicine, University of Bergen, Bergen, Norway; 15 Department of Global Public Health and Primary Care, Neuro-SysMed, Faculty of Medicine, University of Bergen, Bergen, Norway

**Keywords:** Dementia, Pain, Neuropsychiatric symptom, Music intervention

## Abstract

**Background:**

Neuropsychiatric symptoms (NPS) are highly prevalent in nursing home residents. The main aims of this study were to examine whether music-based care (MBC) had sustained effects on NPS in nursing home residents with dementia and chronic pain and to describe the distribution of NPS.

**Methods:**

A secondary analysis of a cluster-randomised controlled trial with intervention and control groups was performed. The 8-week MBC intervention included daily individualised prerecorded music integration. A large sample of nursing home residents with dementia and chronic pain in 12 nursing homes in Norway were screened for pain and dementia by experts and included in the study. The Neuropsychiatric Inventory Nursing Home Edition (NPI-NH) was completed for the residents at pre-test and after the 8-week MBC intervention. Descriptive statistics were used to characterise the sample, and multilevel mixed model analysis assessed the difference in change in NPI-NH scores before and after the intervention between the groups.

**Results:**

The sample (*n* = 232) had a mean age of 86 years (SD 8.8), with 71% being female. Overall, 41%, 38% and 21% had severe, moderate or mild dementia, respectively, while 77% had moderate pain and 23% severe pain. Over two-thirds (68%) of the sample had at least one clinically important (≥ 4) NPI-NH symptom at pre-test, with mean NPI-NH total score of 19 (range 0–89). The most commonly identified NPI-NH subcategories were agitation (44%), affective symptoms (34%) and psychosis (25%) at pre-test. There was no significant difference in changes either comparing the NPI-NH total score (*p* = 0.396) (confidence interval: CI [–2.6 to 6.6]) between the intervention group (*n* = 108) and the control group (*n* = 124) or comparing any of the three NPI subcategories: psychosis (*p* = 0.203) (CI [–0.5 to 2.3]), agitation (*p* = 0.830) (CI [–2.3 to 1.8]) or affective symptoms (*p* = 0.447) (CI [–0.9 to 2.1]).

**Conclusions:**

No statistically significant sustained effect of the MBC intervention on NPI symptoms was found among residents with dementia and chronic pain. Future studies should include measurement points closer to the intervention to evaluate short-term effects of MBC.

**Trial registration:**

Data were collected from June 2020 until June 2021 (ClinicalTrials.gov Identifier: NCT04229446), registered 9/10/2019.

## Introduction

Neuropsychiatric symptoms (NPS) are highly prevalent in those with dementia and can affect up to 90% of nursing home residents with dementia [1, 2]. The main categories of NPS are agitation, emotional symptoms (anxiety and depression) and psychosis (delusions and hallucinations), among others [3]. All of these symptoms can cause distress for nursing home residents with dementia and also impose an emotional burden on other nursing home residents and staff [4–7]. In addition, as shown in another publication based on the same data as our study sample, up to 68% of these residents also report chronic pain, which may exacerbate their condition [8].

Owing to the limited efficacy and serious side effects of psychotropic drugs and analgesics, which are particularly harmful for nursing home residents with dementia, guidelines recommend non-pharmacological interventions for neuropsychiatric symptoms in these individuals [9, 10]. One such intervention is individualised listening to preferred music on a regular basis. However, in a recent randomised controlled trial (RCT), our research group found no prolonged effect of such music-based care (MBC) on chronic pain in residents with dementia in nursing homes [11]. Music therapy falls into two main types: active and passive (receptive). Active therapy engages people in making music—playing instruments, singing or dancing—while passive (receptive) therapy centres on listening, either to live music provided by a therapist or to prerecorded selections [12]. In this study, an 8-week MBC intervention was applied consisting of sessions with individualised prerecorded music (~ 30 min/day) and weekly 45-min group music sessions (8–10 participants) led by a trained musician. The individual sessions used music from Spotify played on a small speaker, and group activities focused on guitar-accompanied singing with occasional rhythmic or vocal exercises.

A Cochrane review from 2018 [13] examined the effects on various health and quality of life-related outcomes of different music-based therapeutic interventions given for at least five sessions to institutionalised people with dementia. The review concluded that individualised music interventions are likely to have some effect on depressive symptoms and improve overall behavioural problems, but may have little or no effect on agitation, aggression or cognition [13]. Another systematic review from 2020 on residents with dementia reported significant reductions in anxiety, depression and apathy after music therapy [14].

Regarding studies that specifically investigated the effect of preferred music listening on NPS, only one paper on a small RCT performed in Italy was identified at the start of the present study. Specifically, Raglio and colleagues compared standard care, therapy by qualified music therapists and listening to music provided by care staff and found no difference among these three groups in behavioural assessments [15]. However, several non-randomised studies have presented the effects of individualised music listening on NPS [16–22].

Against this background, the main aim of this secondary analysis of a previously published randomised controlled study was to assess whether the music intervention resulted in any differences in NPS relative to the findings in the control group in residents with both dementia and chronic pain. The effects of MBC on the overall composition of individual NPS and their prevalence were also described.

## Materials and methods

### Study design

This study is part of a larger study on the effects of MBC on pain in nursing home residents with dementia [8, 11], and reports a secondary analysis from a cluster-randomised trial, originally investigating the effect of MBC on pain (measured using the MOBID-2 Pain Scale) in nursing home patients with dementia and chronic pain [11]. This two-armed trial (randomised complete block design) included 12 nursing homes in Trondheim and Oslo, comprising 36 wards with 645 nursing home residents. Nursing homes were stratified by size, with 18 wards randomised to either the intervention or the control group, totalling 36 wards. The primary researchers collecting the data and performing the analyses were blinded to the group allocation. The intervention was delivered whenever possible and all individuals were analysed on an intention-to-treat basis, regardless of the amount of intervention that they had actually received. We aimed to enrol 120 residents with pain and dementia [11] for each of the control and intervention groups, for a secondary analysis of NPS.

The power calculation was initially conducted in a previous study based on efforts to detect significant findings in the results of MOBID-2 pain assessment as primary analysis [23], and not for the secondary analysis in this study.

### Inclusion criteria and recruitment procedure

**Fig. 1:**
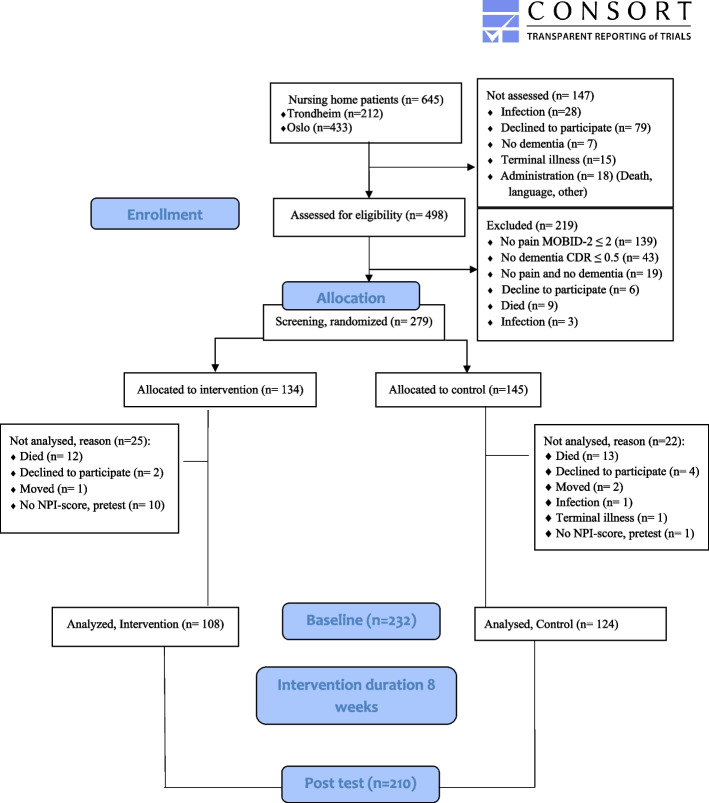
Flowchart of the MBC-study

The detailed original inclusion procedure is described elsewhere [8]. Patients with serious hearing deficits were considered ineligible for participating in this study as the intervention mainly involved listening to music. Of the 645 available nursing home residents, 147 were excluded before screening (79 declined to participate, 28 had infections and 40 lacked dementia or had a short life expectancy). Screening using the Clinical Dementia Rating (CDR) scale [24] and MOBID-2 [25] identified 279 eligible residents with mild to severe dementia and moderate or higher pain intensity. For the current study, we included nursing home residents for whom NPI-NH was filled in at the pre-test. Ultimately, 232 nursing home residents with chronic pain and dementia were included in this study (Fig. 1).

### Music-based care (MBC)

MBC has been developed from neurologic music therapy, with elements from music pedagogy, and is a structured daily care approach rooted in research on music’s impact on cognitive, motor, affective and speech functions [26]. This national programme, anchored in the Ministry of Health in Norway and administered through the Norwegian Resource Centre for Arts and Health, aims to integrate music, song and movement into daily care [27, 28]. MBC has three core elements: (i) individualised music mapping, (ii) systematic use of prerecorded music with documented interventions and (iii) the intervention being delivered by nursing assistants or nurses who know the patient well and have completed the MBC training programme as described below. The individual part of the intervention was delivered using Bluetooth speakers connected to an iPad containing each patient’s individualised playlist. Our study is grounded in a neurobiopsychosocial model, emphasising how MBC interventions engage core neural circuits involved in emotion, memory and motor regulation [29]. These neurobiological mechanisms are hypothesised to downregulate physiological and psychological distress, enhance emotional regulation and promote meaningful engagement.

### Training in MBC and implementation of the intervention

Staff on the wards where the intervention was applied underwent a 3-day, 21-h training programme on MBC, focusing on individualised music mapping, systematic singing, creating positive sound environments, using rhythm for physical activity and integrating music into communication with patients. Guidance was provided on the individualised systematic music mapping, a standardised manual on how to perform mapping for nursing home residents was used prior to implementation and the training covered the documentation of MBC activities. To perform the intervention, staff were required to have attended the entire course and the staff at the nursing home wards were supervised during the study.

In the individual intervention each resident received a daily playlist of prerecorded music, customised based on systematic mapping and assessment of the patient responses. Music selections were categorised, and playlists emphasised music with high durability in interaction between caregiver and resident. Staff in the control wards also underwent the MBC training programme after the 8-week intervention period. During the study period, the control group received the usual care, meaning that they may have listened to music as part of their daily routine in the nursing home. However, they were not exposed to this systematic individualised MBC intervention.

### Music-based care intervention procedure

The 8-week MBC intervention included daily individualised prerecorded music integration (~ 30 min/day) and weekly group music sessions. These group sessions were performed at each centre for 45 min each week for 8–10 participants and were led by a trained music teacher or musician. The sessions primarily involved singing in a group accompanied by a guitar, with occasional physically activating vocal and rhythmic exercises (e.g. clapping or foot stomping). Meanwhile, the integration of individually selected music into daily life involved the selection of familiar music through music mapping, which was then delivered as a Spotify playlist through a small external speaker, preferably with the patient seated in their own room. Headphones were not used, as they could be confusing for patients with progressive dementia. Owing to the COVID-19 pandemic, group sessions were limited in the nursing homes in Oslo, which affected over half of the participants. All MBC activities were recorded in a journal at the nursing wards, specifying the type (care singing, active music listening, group session) and duration. The caregivers leading the intervention received weekly follow-ups for guidance to promote uniformity of the intervention across the wards, support assessments and improve implementation.

### Study procedures and data collection

A team of five registered nurses conducted initial assessments on the nursing home residents, assisted by nursing home personnel familiar with the residents for each data collection instrument. Data were collected before and after the test period in both groups in collaboration with the nursing home staff and physicians. Physicians who were also members of the research team were available for consultation if the nursing home physicians needed guidance. During the baseline testing and clinical examinations, physicians from among the co-authors offered to help optimise pain management strategies before the intervention period for members of both the intervention and control groups. The researchers collecting, analysing and presenting the data were masked to the group allocation throughout the trial. Post-test data collection was performed 2 weeks after the intervention ended. There was an outbreak of COVID-19 infections in Oslo during this period, so post-test data collection there was performed by the primary caregivers who were most familiar with the residents in the nursing home, without the research team in attendance. However, the research team provided guidance for these assessments over the phone.

### Variables

Demographic and clinical data were obtained from the nursing home residents’ medical records. The following data collection instruments were used:

The Clinical Dementia Rating (CDR) scale assesses dementia in older adults across six domains. The total CDR score is calculated using an algorithm that assigns more weight to the memory domain [24]. Scores range from 0 to 3, with a score of 1 or higher being considered to indicate dementia in this study. Scores of 1, 2 and 3 correspond to mild, moderate and severe dementia, respectively. CDR has previously been used in the Norwegian population [2, 30], and the scale has been confirmed to be reliable and valid [24, 31].

Neuropsychiatric Inventory Nursing Home Edition (NPI-NH) is a 12-item inventory that covers the following symptoms: delusion, hallucination, euphoria, agitation/aggression, disinhibition, irritability/lability, depression/dysphoria, anxiety, apathy/indifference, aberrant motor behaviour, night-time behavioural disturbances, and appetite and eating changes.

A score from 0 to 12 is provided for each symptom, which is determined by multiplying severity (score 1–3) by frequency (score 1–4). Three subcategory scores were established, based on a previous principal component analysis [32]: psychosis (the sum-score of delusion and hallucination), agitation (the sum-score of agitation/aggression, disinhibition and irritability) and affective symptoms (the sum-score of depression and anxiety). A score of 4 or higher was defined as indicating a clinically significant symptom for individual items [33]. The NPI-NH has been validated for use in Norwegian nursing homes [34].

The Mobilization-Observation-Behavior-Intensity-Dementia Pain Scale (MOBID-2) for dementia is a two part tool that uses a 0–10 numerical rating scale (NRS) to assess pain in various anatomical regions [25, 35]. Part 1 infers pain intensity from observed musculoskeletal behaviours (like raising arms above the head or bending and stretching the knee and ankle-joints), while Part 2 assesses internal organ, head and skin pain, with scores recorded on pain drawings using schematics of the body. After evaluation, a pain therapist judges the overall score using an NRS scale. MOBID-2 has been shown to be reliable, valid and responsive for assessing pain in patients with severe dementia [36]. In this study, MOBID-2 scores were classified into clinically relevant pain (0–2), moderate pain (3, 4, 5) or severe (≥ 6) pain in the nursing home residents with dementia, where only those in the latter two groups (clinically relevant pain: 3–10) were included in the analysis [11].

### Statistical analysis

The background characteristics of the participants were reported as means and standard deviations for normally distributed continuous variables or as medians and ranges for variables with skewed distributions. Categorical variables, including sex, type of diagnosis, civil status, CDR score and MOBID-2, were described as counts and proportions. Nursing home administrators informed us that the wards within the respective nursing homes were run fairly independently from each other so intervention practices in one ward would be unlikely to spread to other control wards. Block size, determined by the inclusion criteria, was fixed at 12–13 residents for Block 1 and 18–28 residents for Block 2.

A multilevel mixed model analysis with nursing home ward as a random intercept assessed the differences in mean NPI total and subcategory scores before and after the intervention between the groups. The significance level was set at p < 0.05 for the main outcome variables reported in Table 3. IBM SPSS version 24 and STATA version 17 were used for data analysis.

## Results

### Demographics

The total study sample (*n* = 232) had a mean age of 86 years (range 59–103, SD 8.8), with 71% being female. Based on the CDR, 41%, 38% and 21% had severe, moderate and mild dementia, respectively, while 77% had moderate pain and 23% had severe pain at pre-test. There were no statistically significant differences in demographic variables between the intervention (*n* = 108) and control groups (*n* = 124) at the pre-test (Table 1).

**Table 1:** Demographic characteristics and clinical description of the total sample, control group and intervention group at pre-test

	Total sample (*n* = 232)^5^	Control group (*n* = 124)	Intervention group (*n* = 108)	*p*-value
Age, mean (SD)	86.0 (8.8)	84.9 (9.2)	87.3 (8.0)	0.227
Gender				
Females, N (%)	166 (71%)	86 (69%)	80 (74%)	0.427
Civil status				0.329
Married, N (%) Widowed, N (%)	70 (30%) 109 (47%)	38 (31%) 53 (43%)	32 (30%) 56 (52%)	
Dementia diagnosis ^1^				0.921
Dementia sympt. no diagnosis, N (%) Alzheimer, N (%) Vascular, N (%)	169 (72%) 35 (15%) 15 (6%)	91 (73%) 19 (15%) 8 (6%)	78 (72%) 16 (15%) 7 (6%)	
CDR categories ^2^				0.147
Mild (1), N (%) Moderate (2), N (%) Severe (3), N (%)	48 (21%) 89 (38%) 95 (41%)	24 (19%) 42 (34%) 58 (47%)	24 (22%) 47 (44%) 37 (34%)	
CDR Sum of boxes, Mean (SD) ^3^	13.1 (3.8)	13.2 (3.9)	12.9 (3.9)	0.638
MOBID-2 total score ^4^				0.802
Moderate pain, N (%)	180 (77%)	97 (78%)	83 (77%)	
Severe pain N (%)	52 (23%)	27 (22%)	25 (23%)	
NPI-NH total score (Mean, SD)	19.2 (19.3)	19.9 (20.9)	18.2 (17.4)	0.968
Psychosis (Mean, SD)	2.9 (5.1)	3.4 (5.6)	2.5 (4.6)	0.209
Agitation (Mean, SD)	6.9 (8.6)	6.9 (8.5)	6.9 (8.7)	0.915
Affective (Mean, SD)	4.2 (5.8)	4.5 (6.4)	3.8 (5.1)	0.359

*Abbreviations*: *CDR* Clinical dementia rating-scale (0-3), *NH* Nursing Home, *NPI-NH* Neuropsychiatric inventory nursing home edition, *SD* Standard deviation, *sympt* symptoms

*Annotations*:

^1^Dementia diagnosis retrieved from patients’ medical records,

^2^Categories of dementia severity according to CDR where higher numbers refer to more severe dementia

^3^Sum of all individual scores from the CDR rating-scale

^4^MOBID-2 pain scale moderate pain refers to a total assessment score of rating of 3-5, severe pain refers to a MOBID-2 pain scale rating of 6-10

^5^Total sample refers to all included patients with a baseline NPI-score

In the intervention group, the mean total duration of the intervention was 870.7 min (SD 674.6), with a range from 0 to 2655 min, over the 8-week period. The median number of total intervention days was 28, with a range of 0 to 63 days. The 25th, 50th and 75th percentiles regarding the duration of the intervention were 416, 879 and 1421 min, respectively, over the 8-week period. For 27 (25%) patients, no individual music intervention was performed.

Forty-seven residents dropped out of the study from pre-test to post-test for various reasons (Fig. 1). There were no significant differences in sex, level of dementia or level of pain in residents at pre-test compared with those who dropped out after the pre-test (*n* = 47), and those included in the study (Fig. 1). However, there was a statistically significant difference in age and those who dropped out were older than those who remained (*p* = 0.012).

### Prevalence and distribution of NPS

As outlined in Table 1, there was no significant difference between the intervention and control groups regarding the NPI-NH total score (*p* = 0.968) at the pre-test. There were also no significant differences in the NPI-NH subcategories of psychosis, agitation and affective symptoms.

More than two-thirds (68%) of the residents had at least one clinically important (≥ 4) NPI-NH symptom at pre-test, with a mean NPI-NH total score of 19.2 (range 0–89). As outlined in Table 2, the most common NPI-NH subcategory scores (with at least one individual symptom ≥ 4) were agitation (44%, mean 6.9), affective symptoms (34%, mean 4.5) and psychosis (25%, mean 3.4) at pre-test (Table 2). As shown in Table 2, the most common clinically significant (≥ 4) individual NPI-NH items were irritability (37%), agitation (29%), anxiety (25%), depression (22%) and delusions (21%).

**Table 2:** Description of clinical variables and proportions of any clinically significant symptom (NPI-NH ≥ 4) and any symptom (NPI-NH ≥ 1) from the NPI total score, sub-categories, and individual items at baseline

	Total sample (*n* = 232) *		Control group (*n* = 124)		Intervention group (*n* = 108)	
Major NPI-NH categories	NPI ≥ 4 N (%)^1^	Any symptom N %^2^	NPI ≥ 4 N %^1^	Any symptom N %^2^	NPI ≥ 4 N %^1^	Any symptom N %^2^
Total score (range 0–89)	158 (68.1%)	200 (86.2%)	84 (67.7%)	108 (87.1%)	74 (68.5%)	92 (85.2%)
Psychosis (range 0–24)	59 (25.4%)	97 (41.8%)	33 (26.6%)	53 (42.7%)	26 (24.1%)	44 (40.7%)
Agitation (range 0–36)	102 (43.9%)	146 (62.9%)	57 (45.9%)	79 (63.7%)	45 (41.7%)	67 (62.0%)
Affective (range 0–24)	79 (34.0%)	123 (53.0%)	42 (33.9%)	69 (55.7%)	37 (34.2%)	54 (50.0%)
NPI-NH sub-categories						
Delusions	49 (21.1%)	78 (33.6%)	28 (22.6%)	43 (34.7%)	21 (19.4%)	35 (32.4%)
Hallucinations	24 (10.3%)	51 (21.9%)	15 (12.1%)	28 (22.6%)	9 (8.3%)	23 (21.3%)
Agitation	66 (28.5%)	97 (41.8%)	30 (24.2%)	46 (37.1%)	36 (33.3%)	51 (47.2%)
Anxiety	57 (24.6%)	90 (38.8%)	31 (25.0%)	50 (40.3%)	26 (24.1%)	40 (37.0%)
Depression	51 (21.9%)	86 (37.1%)	28 (22.6%)	51 (41.1%)	23 (21.3%)	35 (32.4%)
Euphoria	13 (5.6%)	19 (8.2%)	10 (8.1%)	15 (12.1%)	3 (2.8%)	4 (3.7%)
Disinhibition	37 (15.9%)	64 (27.6%)	21 (16.9%)	37 (29.8%)	16 (14.8%)	27 (25.0%)
Irritability	86 (37.1%)	125 (53.9%)	46 (37.1%)	63 (50.8%)	40 (37.0%)	62 (57.4%)
Abb. motor behaviour	37 (15.9%)	50 (21.6%)	20 (16.1%)	27 (21.8%)	17 (15.7%)	23 (21.3%)
Sleep	29 (12.5%)	43 (18.5%)	13 (10.5%)	19 (15.3%)	16 (14.8%)	24 (22.2%)
Appetite	27 (11.6%)	31 (13.4%)	11 (8.9%)	13 (10.4%)	16 (14.8%)	18 (16.7%)
Apathy	35 (15.1%)	60 (25.9%)	ta23 (18.5%)	39 (31.5%)	12 (11.1%)	21 (19.4%)

*Abbreviations*: *NPI-NH* Neuropsychiatric inventory nursing home edition,

^*^Total sample refers to all included patients with a baseline NPI-score

^1^The presence of any clinically significant NPI-symptom (≥ 4)

^2^The presence of any NPI-symptom

### Effect of MBC on NPS

In total, we analysed 108 residents in the intervention group and 124 in the control group. Our data did not reveal any statistically significant differences between the intervention and control groups regarding changes in NPI from pre-test to post-test. The estimated between-group difference in change was 1.6 (95% CI [–2.6 to 6.6], *p* = 0.396, ES = 1.99), as shown in Table 3.

**Table 3:** The effect of the music-based caregiving intervention and between group differences in points from pre to post-test in NPI-NH total score and NPI-NH sub-categories

	Control group (*n* = 124)			Intervention group (*n* = 108)s		Between group differences^1^		*p*-value ^2^
	Mean	(95% CI)		Mean	(95% CI)	Mean	(95% CI)	
NPI NH^3^ total score			NPI NH^3^ total score					
Pre-test (*n* = 124)	19.9	16.2, 23.7	Pre-test (*n* = 108)	18.2	14.9, 21.5			
Post-test (*n* = 109)	17.4	14.4, 20.4	Post-test (*n* = 101)	18.1	14.5, 21.6			
Change from pre-test	−1.1	−4.9, 2.7	Change from pre-test	0.5	−1.9, 3.0	1.6	−2.6, 6.6	0.396
Psychosis			Psychosis					
Pre-test	3.4	2.4, 4.3	Pre-test	2.5	1.7, 3.4			
Post-test	2.7	1.8, 3.5	Post-test	2.7	1.7, 3.8			
Change from baseline	−0.7	−1.8, 0.3	Change from baseline	0.2	−0.8, 1.1	0.9	−0.5, 2.3	0.203
Agitation			Agitation					
Pre-test	6.9	5.4, 8.4	Pre-test	6.9	5.2, 8.5			
Post-test	6.7	5.2, 8.2	Post-test	6.5	4.8, 8.3			
Change from baseline	−0.01	−1.6, 1.7	Change from baseline	−0.3	−1.5, 0.9	−0.2	−2.3, 1.8	0.830
Affective			Affective					
Pre-test	4.5	3.4, 5.7	Pre-test	3.8	2.8, 4.8			
Post-test	3.5	2.6, 4.4	Post-test	3.4	2.4, 4.5			
Change from baseline	−0.9	−2.1, 0.2	Change from baseline	−0.3	−1.4, 0.7	0.6	−0.9, 2.1	0.447

*Abbreviations*: *NPI-NH* Neuropsychiatric inventory nursing home edition, *95% CI* 95% confidence interval

^1^Mean difference between the intervention group and control group, lower numbers favour the intervention

^2^Results from a mixed model analysis

^3^NPI-NH total score where lower scores indicate less symptom burden

Furthermore, as outlined in Table 3, there was no significant difference in change in NPS-NH between before and after the intervention, comparing the NPI-NH total score (1.6, 95% CI [–2.6 to 6.7]) in the intervention group to the control group. There were also no significant differences for any of the three subcategories of psychosis (0.9, 95% CI [–0.5 to 2.3]), agitation (–0.2, 95% CI [–2.3 to 1.8]) and affective symptoms (0.6, 95% CI [–0.9 to 2.1]). Sensitivity analysis revealed that there was no significant correlation between the total duration of intervention and the change in NPI scores from pre-test to post-test.

## Discussion

This secondary analysis found that MBC did not produce a sustained effect on the total NPI score or on the scores of any of its three main subcategories—psychosis, agitation and affective symptoms—among Norwegian nursing home residents with dementia and chronic pain. Additionally, more than two-thirds of the participants had neuropsychiatric symptoms at pre-test, and agitation was the most common single symptom.

The finding of no effect of the MBC intervention on NPS over 8 weeks is in accordance with the findings of the previously mentioned RCT [16] and three recently reported RCTs [37–39]. For example, McCreedy et al. [37] analysed 976 residents in US nursing homes with dementia who listened to personalised music daily. In that study, personalised music intervention was not significantly effective in reducing agitated behaviours or the use of psychotropic drugs after 4 months [37]. In that study, pragmatic inclusion of residents from 54 nursing homes was performed, while the present study used strict diagnostic inclusion criteria for both dementia and chronic pain.

Another RCT by Kwak et al. [38] using a randomised cross-over design included 59 nursing home residents with dementia from 10 nursing homes and investigated the effect of music listening on agitation, behaviour and psychotropic drug use over 14 weeks. Although a positive trend was seen, the intervention did not confer statistically significant benefits for any of these variables [38].

The aim of the most recent RCT, which included 158 people with dementia from nursing homes in the Netherlands, was to evaluate the effects of an individual music therapy intervention and an individual music listening intervention on neuropsychiatric symptoms and quality of life [39]. The results revealed that hyperactive behaviour as assessed by NPI-NH was significantly reduced for those in the individual music listening intervention group at follow-up, and that restless behaviour was significantly reduced in this group at post and follow-up measurements, compared with those in the control group. In addition, no significant differences were found between the groups regarding the other NPI-NH subcategories.

Meanwhile, various observational studies have reported associations between individual music listening and a wide range of behavioural and psychological symptoms in residents with dementia in nursing homes [22, 40–43]. Among these is a recently reported large observational study involving 98 nursing homes and nearly 26,000 residents, which described that the proportion of residents with dementia who discontinued antipsychotic medication use over a 6-month period was associated with individualised music listening [44].

Apart from the present study, to the best of our knowledge only one previous study has investigated the effects of preferred music listening on patients with NPS, dementia and pain [45]. In that study, which used both qualitative and quantitative methods, 113 care home residents received 30 min of daily preferred music listening for 3 weeks. The results revealed that listening to music significantly reduced anxiety and depression in those without pain, while patients with severe pain did not benefit from the intervention. These results indicate that residents with both dementia and pain may be more refractory to the effects of listening to music on NPS symptoms than residents with dementia but no pain.

The studies cited above examined the sustained effects of daily music listening, with assessments conducted days after the intervention. However, some studies have investigated the immediate effects of individual music listening in nursing homes, measured during or shortly after the session [18, 40, 46]. For example, two studies found benefits from 30 min of listening to music 1–3 times weekly, although no reduction in aggression was observed when behaviour was assessed at the time of listening [18, 40]. Moreover, an RCT of 90 nursing home patients with dementia found that individual music listening reduced behavioural and psychological symptoms during and immediately after the session, but the effects were temporary [46].

Concerning the prevalence and types of individual NPS and how they are affected by MBC, the finding from this study that nearly 68% of nursing home residents with dementia had any clinically important NPS from the NPI-NH at pre-test is similar to the results of previous studies [1, 2, 4, 47]. Upon comparing the mean sum scores of NPI-NH at baseline, a previous study found a score of 14.9 in a general NH population [4], while a similar study by Selbæk et al. [1] found one of 18.2 in nursing home residents with dementia. Our sample had a higher mean total NPI-NH score (19.1) at pre-test, but that may have been due to our included population also suffering from chronic pain. Previous studies have reported significant reductions in NPS such as agitation and depression as a result of interventions involving pain treatment [48, 49].

Regarding the NPI subcategories, agitation (44%) was most common in the present study, followed by affective symptoms (34%) and psychosis (25%). When looking at mean values in other samples of Norwegian nursing home residents, agitation had the highest mean score, followed by affective symptoms and psychosis [1, 4]. In the general NH population, a study reported mean scores for the subcategories of agitation, affective symptoms and psychosis of 4.5, 3.9 and 1.9, respectively. Meanwhile, a study on nursing home patients specifically with dementia reported higher mean scores for agitation (mean 6.5) and psychosis (mean 3.2), but not for affective symptoms (mean 3.2) [1]. Our study investigating nursing home residents with both dementia and pain found even higher scores for agitation (6.9), affective symptoms (4.2) and psychosis (2.9) compared with those in the general NH population. Our results may support the notion that pain can increase NPS in nursing home residents with dementia.

Among the 12 individual NPI symptoms, irritability (37%), agitation (29%), anxiety (25%), depression (22%) and delusions (21%) were the most prevalent, which is similar to the findings in previous studies where irritability, depression, anxiety, agitation and delusions were among the most common NPS symptoms in NH populations [1, 4, 47].

Although this study was conceptually informed by a neurobiopsychosocial perspective, the hypothesised mechanisms were not directly tested. Recent neuroimaging and neurobiological studies have demonstrated that personalised and emotionally salient music can engage neural networks involved in autobiographical memory, reward processing and emotional regulation in older adults, including those with cognitive impairment [29, 50, 51]. This work also suggests that such mechanisms are highly state- and context-dependent, requiring sufficient emotional engagement and intervention intensity to be consistently activated. Furthermore, such studies are mechanistic in nature, often conducted in cognitively healthy samples, and do not account for the neurobiological changes associated with dementia, nor for issues of dose, feasibility or sustained clinical outcomes. This highlights the importance of large pragmatic trials in clinical populations to establish real-world effectiveness of music-based interventions for persons with dementia in nursing homes.

COVID-19 restrictions that were implemented throughout Norway introduced a bias specific to the group-format MBC. As the individual MBC could proceed as planned, components driven by individualised stimulation were less affected by these restrictions. To examine a possible effect of COVID-19, we entered ‘city’ (Trondheim vs. Oslo) into the regression model. This covariate was not statistically significantly different. Additionally, owing to the outbreak of COVID-19 during the intervention period in Oslo, the nursing homes that were affected needed to extend the duration of the intervention period by approximately 1 week, as patients at these nursing homes lost some intervention days during the original study period. However, it was not considered that this markedly influenced the obtained results.

Among the strengths of the present study are the relatively large homogeneous sample and the use of a validated instrument with high reliability in Norwegian nursing homes. In addition, the well-organised inclusion procedure in nursing homes ensured that the sample consisted of NH residents with both dementia and chronic pain. One limitation of this work was that the study analysed the secondary outcomes of a previously published study and was not powered for the context of the present study. Another limitation is that we did not measure the direct effect of the intervention but instead performed measurements after the intervention had been completed. A third limitation was that the pre-test data were collected before contact and collaboration with the nursing home physician, which also included a review of the pharmacological treatment. Optimisation of analgesic prescriptions was performed, but only in five patients, without knowing which patients were randomised to the two study groups; however, the small effect of this should have been distributed across the groups. The variability in the exposure time of the intervention was also a limitation of the present study. To ensure that the analyses reflect the real-world effectiveness of the intervention, the intention-to-treat principle was followed. Additional research would benefit from explicitly evaluating feasibility and implementation outcomes of MBC of this kind, such as staff workload, organisational readiness and intervention fidelity, alongside clinical endpoints, to obtain a better understanding of when and how music-based interventions (with different types of music) can be sustainably integrated into routine care. The same would also be true for dose–response considerations where the observed median dose and variability should be interpreted descriptively. Future trials should further incorporate measurements of variables reflecting engagement with the intervention, such as affective response, autobiographical recall or social interaction, alongside dose and fidelity metrics, and the associations of such variables with the hypothesised mechanisms.

## Conclusion

The residents of nursing homes in Norway included in the present study had both dementia and chronic pain, and more than two-thirds had NPS at the pre-test. Unfortunately, the MBC intervention did not have a sustained effect on their NPS. The absence of sustained effects underscores the need for future research to employ explicit conceptual frameworks that integrate intervention components, dose, delivery conditions and mechanistic outcomes. Despite the lack of significant findings in this study, the possibility of various music interventions having beneficial effects on NPS should not be ruled out. It is particularly beneficial if such interventions can be offered on an individual basis by staff who are involved in their care on a regular basis. However, our findings suggest the value of evaluating the intervention during or immediately after it has been delivered in future research.

## Data Availability

The datasets used and/or analysed during the current study are available from the corresponding author on reasonable request.

## Abbreviations

CDR: Clinical Dementia Rating scale
CI: Confidence interval
COVID-19: Coronavirus disease 2019
ES: Effect size
MBC: Music-based care
MOBID: Mobilization-Observation-Behavior-Intensity-Dementia assessment form
NH: Nursing home
NPI-NH: Neuropsychiatric Inventory, Nursing Home Edition
NPS: Neuropsychiatric symptom
RCT: Randomised controlled trial
SD: Standard deviation
